# A New Digital Assessment of Mental Health and Well-being in the Workplace: Development and Validation of the Unmind Index

**DOI:** 10.2196/34103

**Published:** 2022-01-17

**Authors:** Anika Sierk, Eoin Travers, Marcos Economides, Bao Sheng Loe, Luning Sun, Heather Bolton

**Affiliations:** 1 Unmind Ltd London United Kingdom; 2 The Psychometrics Centre Judge Business School University of Cambridge Cambridge United Kingdom

**Keywords:** mental health, well-being, mHealth, measurement

## Abstract

**Background:**

Unmind is a workplace, digital, mental health platform with tools to help users track, maintain, and improve their mental health and well-being (MHWB). Psychological measurement plays a key role on this platform, providing users with insights on their current MHWB, the ability to track it over time, and personalized recommendations, while providing employers with aggregate information about the MHWB of their workforce.

**Objective:**

Due to the limitations of existing measures for this purpose, we aimed to develop and validate a novel well-being index for digital use, to capture symptoms of common mental health problems and key aspects of positive well-being.

**Methods:**

In Study 1A, questionnaire items were generated by clinicians and screened for face validity. In Study 1B, these items were presented to a large sample (n=1104) of UK adults, and exploratory factor analysis was used to reduce the item pool and identify coherent subscales. In Study 2, the final measure was presented to a new nationally representative UK sample (n=976), along with a battery of existing measures, with 238 participants retaking the Umind Index after 1 week. The factor structure and measurement invariance of the Unmind Index was evaluated using confirmatory factor analysis, convergent and discriminant validity by estimating correlations with existing measures, and reliability by examining internal consistency and test-retest intraclass correlations.

**Results:**

Studies 1A and 1B yielded a 26-item measure with 7 subscales: *Calmness*, *Connection*, *Coping*, *Happiness*, *Health*, *Fulfilment*, and *Sleep*. Study 2 showed that the Unmind Index is fitted well by a second-order factor structure, where the 7 subscales all load onto an overall MHWB factor, and established measurement invariance by age and gender. Subscale and total scores correlate well with existing mental health measures and generally diverge from personality measures. Reliability was good or excellent across all subscales.

**Conclusions:**

The Unmind Index is a robust measure of MHWB that can help to identify target areas for intervention in nonclinical users of a mental health app. We argue that there is value in measuring mental ill health and mental well-being together, rather than treating them as separate constructs.

## Introduction

### Background

Poor mental health affects hundreds of millions of people worldwide, impacting individual quality of life and creating a significant economic burden for employers [1-3]. With evidence that many mental health problems are preventable or treatable [4-6], there is a strong business case for employers to invest in preventative mental health solutions for their workforces [7,8]. In recent years, desktop and mobile health (mHealth) apps have begun to fulfill this preventative remit. Digital technologies might be particularly useful in a workplace setting, where traditional reactive approaches tend to have low uptake [9].

Unmind is a workplace, digital, mental health platform providing employees with tools to help them track, maintain, and improve their mental health and well-being (MHWB) and allowing employers to gain insight into the overall well-being of their employees through anonymized, aggregated data. Consistent with the contemporary understanding of mental health as a complete state of physical, mental, and social well-being [10], the Unmind approach encourages users to take a holistic approach to understanding and managing their MHWB. This holistic approach may be particularly relevant for promoting regular, proactive use of the platform in working adults.

Measurement plays a key role on the Unmind platform. First, given the broad range of content available on the platform, it is important to guide users toward the materials best suited to their particular needs. Second, allowing users to monitor and reflect on their own mental health has been shown to improve engagement with mHealth apps [11,12]. Finally, there is some evidence that measurement tools may directly improve users’ mental health, perhaps by encouraging them to reflect upon their own mental states [13,14]. The Insights section of the Unmind platform consists of 2 tools: a brief Check-In (mood tracker) and the more in-depth Unmind Index. In this article, we describe the development and validation of the Unmind Index.

### The Case for a Novel Measure

There is a distinction between mental health (the absence of mental illness) and mental well-being. Existing self-report scales are typically intended to measure one or the other factor. On the one hand, diagnostic mental health measures are used in clinical practice to help diagnose patients with specific mental health disorders (as described in the Diagnostic and Statistical Manual of Mental Disorders [DSM]-V or International Classification of Diseases [ICD]-11). On the other hand, positive mental well-being scales are intended to measure broader well-being and quality of life and are typically based on principles from positive psychology. Although distinct, these 2 factors are strongly correlated [15]. Ideally, the self-monitoring features of an mHealth app should capture both factors.

As they are, existing diagnostic and positive mental well-being scales have strengths and weaknesses for use in mHealth apps. Diagnostic scales provide sensitive, well-validated measures of specific aspects of mental ill-health, such as the Patient Health Questionnaire 9 (PHQ-9; depression) [16], General Anxiety Disorder 7 (GAD-7; anxiety disorders) [17], or the Insomnia Severity Index (ISI) [18]. However, these scales are a poor fit for a digital mental health platform for 2 reasons.

First, by design, these scales focus on disorder-specific symptoms. For example, the GAD-7 will assess the extent to which anxiety impairs an individual's day-to-day life but will not directly assess their ability to relax or remain calm under usual circumstances. As a result, these scales typically have excellent sensitivity for users with poor mental health but inadequate sensitivity for healthier users who would not be seen in a clinical setting. This is also reflected in the language typically used in diagnostic tests, which is necessarily problem-focused. Presenting users with a large number of negatively phrased questions is likely to discourage user engagement in a digital mental health platform, and these questions may feel less relevant to healthier users.

Second, it is widely recognized that many mental health disorders are strongly interrelated, with largely overlapping symptoms. It has been shown that much of the variance across a broad range of mental health scales is explained by a single latent factor capturing participants’ overall state of mental health or well-being [19]. Individual diagnostic scales are not designed to measure this higher-order MHWB factor, and although it could be approximated by averaging scores across diagnostic scales for different disorders, this approach has not been validated.

Holistic scales intended to assess overall mental well-being address both of these limitations. These scales are typically designed using positive psychology principles, use positive language, are calibrated to measure the range of mental health seen in the general population, and capture a broader range of mental health–related constructs than diagnostic tests can. Holistic scales include the Warwick-Edinburgh Mental Wellbeing Scales (WEMWBS) [20] and the Brief Inventory of Thriving (BIT) [21]. However, these scales do not reliably measure the various components of mental health, such as happiness, social support, or sleep quality, and so are of limited use for guiding users to appropriate content or for self-reflection.

### Goals for the Unmind Index

Given the limitations of existing measures for our purposes, we decided to develop a new measure for use on the Unmind platform. Five primary goals guided the development of this measure. First, we decided to combine items that measure mental health and those that measure well-being. That is, we aimed to measure MHWB as a combined construct. Second, the Unmind Index was intended to measure the different subdomains of MHWB (eg, social functioning, mood, anxiety), providing users with personalized feedback and actionable content recommendations. Third, it was also intended to provide a single overall MHWB score, combining scores from the individual subdomains in a scientifically validated way. Fourth, the Unmind Index was intended to empower users to monitor their mental health over time, spotting trends. Finally, as a workplace platform, the Unmind Index was intended to allow employers to access their employees’ aggregated data to understand trends and inform their well-being strategy. Beyond these goals, we sought to create a measure that was brief enough to encourage regular completion by casual users of the Unmind platform, easy to complete with minimal instruction, and targeted to nonclinical (workplace) populations.

**Figure 1:**
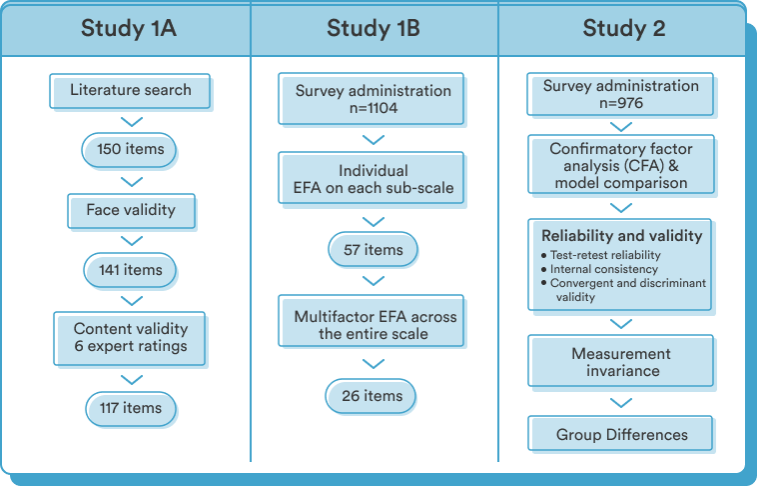
Overview of the structure of Studies 1A (scale development), 1B (exploratory factor analysis), and 2 (validation). EFA: exploratory factor analysis.

This paper reports the development and validation of the Unmind Index in 3 parts. Study 1A described the generation of candidate items and the assessment of their validity. Study 1B documented the item selection process and the identification of the various facets of MHWB to be captured by the Unmind Index, using exploratory factor analysis (EFA). Finally, Study 2 described the validation of the Unmind Index, including confirmatory factor analysis (CFA) to identify the appropriate approach to calculating the overall MHWB score. It also demonstrated the psychometric properties of the Unmind Index and its convergent validity with existing diagnostic and holistic measures. It also established discriminant validity against measures of personality, documented measurement invariance, and explored gender and age differences in scores (see Figure 1 for an overview).

### Ethics

The study received ethical approval from the University of Cambridge (Judge Business School Departmental Ethics Review Group, approval number 20-061). All participants provided informed consent prior to taking part.

## Study 1A: Scale Development

### Item Generation and Face Validity

An initial pool of 150 items was created by an experienced UK-trained clinical psychologist (HB) for the proposed 7 constructs underpinning our conceptualization of MHWB. The constructs were named *Happiness* (37 items), *Calmness* (20 items), *Coping* (15 items), *Health* (10 items), *Sleep* (8 items), *Energy* (7 items), and *Vitality* (44 items). All items were presented to 4 nontechnical members of staff at Unmind who were asked to assess each item for face validity [22] by providing qualitative feedback on the semantic clarity of each item. Based on this feedback, 5 items were reworded, and 9 items were discarded. The remaining pool of 141 items was reviewed and edited by a professional copywriter to improve readability and tone of voice.

### Content Validity

A panel of 6 UK-trained clinical psychologists (4 female, 2 male), with a mean 14.3 (range 12-20) years of experience in adult mental health, were individually asked to rate each of the remaining items with respect to how well it assessed the defined construct it purported to measure (1=not relevant, 2=somewhat relevant, 3=quite relevant, 4=highly relevant). They also provided further qualitative feedback on content validity and suggestions for item rewording where applicable. Interrater reliability was assessed via the item content validity index (I-CVI), and items with an I-CVI <.8 were removed—a benchmark considered to present an excellent strength of agreement between raters [23]. Based on the experts’ suggestions regarding item wording, we added in 9 slightly reworded items in addition to their original equivalent. The resulting final pool of 117 candidate items was then explored in an EFA study, described next.

## Study 1B: Exploratory Factor Analysis

### Methods

#### Participants

We recruited a convenience sample of UK-based adults (n=1180). The sample size was determined based on a commonly accepted item-to-variable ratio of 1:10 [24,25], with 117 items. Individuals were recruited via the online recruitment platform Prolific [26] and invited to participate in an online survey built using the Gorilla Experiment Builder [27]. Prolific has been empirically tested across key attributes such as participant response rates and data quality [28]. Upon joining the Prolific participant pool, individuals are required to complete an extensive prescreening questionnaire designed to help researchers automatically screen for eligibility criteria at the recruitment stage. Participants were eligible for the study if they were aged 18-65 years, based in the United Kingdom, proficient in English, and recently active on the Prolific platform. To increase sample representativeness, the research team stratified the study population with regard to sex and ethnicity (according to the UK census data from 2011) and recruited each strata using separate study advertisements that were identically worded. Informed consent was obtained from all participants, and they received monetary compensation for their participation. Each participant was instructed to respond to 117 candidate items and a demographics questionnaire.

Of the 1180 participants that completed the study, 76 were excluded in total, leaving 1104 participants in the final analysis. Of these, 7 completed the study faster than our minimum required time threshold of 5 minutes, 3 reported not responding honestly, and 66 answered with only 1 response option in the Unmind Index. Some of the excluded participants met more than one of these criteria. Mean age was 40.0 (SD 9.8) years, with 49.8% (550/1104) of participants identifying as female, 49.8% (550/1104) as male, and 0.4% (4/1104) as other. Regarding ethnicity, 6.9% (77/1104) participants identified as Asian/Asian-British, 3.1% (34/1104) as Black/African/Caribbean/Black British, 2.1% (23/1104) as Mixed, 0.8% (9/1104) as Other, and 87.1% (961/1104) as White.

#### Measures

The Unmind Index uses a reporting period of the past 2 weeks. Respondents are shown the prompt “During the past two weeks I have...”, followed by the item text (eg, “been feeling cheerful or bright in my mood”) and are asked to rate how often each item applies to them on a 6-point Likert scale from “No days” (0) to “Every day” (5). A 6-point scale was chosen as previous evidence suggests that middle response options are often misinterpreted by respondents and can encourage deviation to the mean [29,30]. To ensure the final Unmind Index would be brief enough to encourage regular completion by users of the Unmind platform, we committed to an upper limit of 29 items in total, with a minimum of 3 items per construct (based on recommendations by Hair and colleagues [31]).

#### Statistical Analysis

We took a 2-step data-driven approach to selecting items to include in the Unmind Index. In the first step, we performed single-factor EFA for each of the 7 subscales (*Happiness*, *Calmness*, *Coping*, *Health*, *Sleep*, *Energy*, and *Vitality*) separately and removed items with factor loadings <.7 (a stringent cut-off). This step was repeated iteratively for each subscale until a satisfactory set of items remained for each factor. All EFA analyses used the psych package for R [32].

In the second step, we combined the items identified in the first step and performed a multifactor EFA. As the various subscales were expected to be related, we used an oblimin rotation. To ensure the data were suitable for factor analysis, we assessed the Bartlett test of sphericity and the Kaiser-Meyer-Olkin test of sampling adequacy, with .5 taken as the minimal acceptance level [33]. The number of factors to retain was determined using Horn parallel analysis with 5000 iterations [34], implemented in the paran package for R [35]. Items that did not load on any factor with a loading >.4 were dropped at this stage.

Given the primary purpose of the Unmind Index is to direct users to content on the Unmind platform, it was decided that the factor structure of the Unmind Index should mirror the structure of this content wherever possible. For this reason, we made minor changes to the factor structure identified by EFA to accommodate these theoretical and practical constraints.

Finally, to test whether it was appropriate to combine the factors identified at this stage into a single overall MHWB score, we examined the proportion of variance in the final items selected that could be explained by a single-factor model.

### Results

Using the iterative, single-factor EFA procedure outlined in the previous section, the item pool was reduced from 118 items to 57 items across the 7 scales. The Kaiser-Meyer-Olkin measure of sampling adequacy for the reduced item pool was high at .99, and the Bartlett test of sphericity was significant (χ^2^_56_= 62376.6, *P*<.001), indicating the items were appropriate for factor analysis. We then performed multifactor factor analysis on this pool of 57 items. Parallel analysis revealed that the eigenvalues of the randomly generated data were exceeded by the first 9 eigenvalues in our data set, and thus, 9 factors were extracted and rotated.

Of these factors, 5 corresponded to our predefined constructs of *Happiness*, *Coping*, *Health*, and *Sleep*. Items intended to assess calmness loaded onto 2 separate factors, 1 reflecting somatic feelings of tension (*Tension*) and 1 reflecting the cognitive experience of worrying (*Worry*). We combined these to form a single factor, *Calmness*. Items intended to measure the *Vitality* construct loaded onto multiple factors: 1 reflecting interpersonal relationships (*Connection*), 1 relating to meaning and purpose in life (*Purpose*), and 1 relating to a sense of achievement or accomplishment (*Achievement*). On practical grounds, we retained the *Connection* factor and combined *Purpose* and *Achievement* to create a new factor, *Fulfilment*. None of the factors identified reflected the predefined *Energy* construct, and items intended to measure this construct either did not load on any factor or loaded weakly on *Happiness*, *Health*, or *Fulfilment*. We therefore did not include *Energy* as a subscale. At this point, we excluded 31 items with factor loadings <.4.

Following these changes, 26 items remained in the Unmind Index, measuring 7 factors. These factors were *Happiness* (5 items), *Calmness* (4 items), *Coping* (3 items), *Sleep* (3 items), *Health* (3 items), *Connection* (3 items), and *Fulfilment* (5 items). Finally, there were substantial positive correlations between all factors, and we found that a single factor could explain 51.9% of the variance in these 26 items, indicating that combining factor scores to obtain a total would be appropriate.

## Study 2: Scale Validation

### Methods

#### Participants

To validate the Unmind Index developed in Study 1, a new sample of participants (n=1000) was recruited via the Prolific platform. Inclusion criteria were equivalent to Study 1. The sample composition was representative of the UK population with respect to age, sex, and ethnicity (a feature developed by Prolific but not yet available at the time of Study 1). To recruit a nationally representative sample, Prolific utilizes participants’ prescreening responses to stratify their participant pool. Based on guidelines from the UK Office of National Statistics, age is stratified into 5 bands of 9 years each (18-27, 28-37, 38-47, 48-57, and ≥58 years), sex into male and female, and ethnicity into 5 categories (Asian, Black, Mixed, Other, and White), resulting in 50 subgroups. Using 2011 UK census data, Prolific automatically calculates the proportion of each subgroup in the UK national population and allocates participants accordingly.

Mean reported age was 46.1 (SD 15.7) years, with 51.2% (500/976) of participants identifying as female, 48.7% (475/976) identifying as male, and 1 identifying as Other. For ethnicity, 84.8% (828/976) identified as White, 7.1% (69/976) as Asian/Asian British, 3.8% (37/976) as Black/African/Caribbean/Black British, 2.5% (24/976) as Mixed, and 1.8% (18/976) as Other. To examine test-retest reliability, 250 participants were asked to repeat the new measure 1 week later, of whom 240 completed the follow-up. Mean age of the retest group was 48.1 (SD 15.5) years; 49.2% (118/240) of participants identified as female, and 50.8% (122/240) identified as male. For ethnicity, 86.7% (208/240) identified as White, 5.8% (14/240) as Asian/Asian British, 3.3% (8/240) as Black/African/Caribbean/Black British, 2.9% (7/240) as Mixed, and 1.3% (3/240) as Other.

#### Measures

Participants responded to the 26-item Unmind Index developed in Study 1, with items presented in randomized order. They also completed a demographics questionnaire matching the one that was used in Study 1B and a battery of existing self-report measures to allow for testing of convergent and discriminant validity for each well-being subconstruct. Each existing measure was expected to correlate positively or negatively with 1 Unmind Index subscale or with the overall Unmind Index score. The external measures used are summarized in Table 1.

**Table 1:** Convergent and discriminant validity measures used in Study 2.

Measure	Label/abbreviation	Domain	Items	Subscales	Response options	Score range	Reliability (α)	Unmind Index subscale
Patient Health Questionnaire 9 [16]	PHQ-9	Depression	9	-^a^	4	0-27	.90	Happiness
General Anxiety Disorder 7 [17]	GAD-7	Anxiety	7	-	4	0-21	.93	Calmness
Hospital Anxiety and Depression Scale [36]	HADS	Anxiety, depression	14	Anxiety, Depression	4	0 - 21	.90 (Anxiety), .86 (Depression)	Calmness (Anxiety), Happiness (Depression)
Perceived Stress Scale [37]	PSS	Stress	10	-	5	0-40	.92	Coping
Insomnia Severity Index [18]	ISI	Sleep disorders	7	-	4	0-28	.91	Sleep
Revised UCLA Loneliness Scale [38]	ULS-20	Loneliness and social isolation	20	-	4	20-80	.95	Connection
PROMIS^b^ Global Health [39]	PROMIS-10	Mental, physical, and overall health	10	Metal health, Physical health, Combined health	5^c^	4-20 (subscales); 10-50 (combined)	.85 (Mental), .71 (Physical), .88 (Combined)	Health (PROMIS Physical)
Brief Inventory of Thriving [21]	BIT	Positive well-being	10	-	5	1-5	.93	Fulfilment
Warwick-Edinburgh Mental Well-being Scale [20]	WEMWBS	Overall well-being	14	-	5	14-70	.95	Total score
Ten-Item Personality Inventory [40]	TIPI	Big five personality traits	10	Extraversion, Agreeableness, Conscientiousness, Emotional stability, Openness	7	2-14	.77 (Extraversion), .46 (Agreeableness), .66 (Conscientiousness), .77 (Emotional stability), .42 (Openness)	None (control measure)

^a^The measure does not have subscales.

^b^PROMIS: Patient-Reported Outcomes Measurement Information System.

^c^PROMIS-10 includes a 10-point pain scale that was recoded to a 5-point scale.

#### Statistical Analysis: Confirmatory Factor Analysis

All statistical analyses were performed in R [41]. To assess the factor structure of the Unmind Index, we compared a variety of possible CFA models: a correlated factors model, a bifactor model, and a second-order model. Models were fit using the lavaan package for R [42] using maximum-likelihood estimation with robust Huber-White standard errors and fit statistics. In all models, each of the 26 items loads onto 1 of 7 Unmind Index subscales (*Happiness*, *Sleep*, *Coping*, *Calmness*, *Health*, *Connection*, and *Fulfilment*) in line with the results of the EFA reported in the previous section.

Models differed in how the relationship between these subscales was conceptualized. In the correlated factors model, the full covariance between each subscale is modelled explicitly. This approach can provide a flexible fit to the data but is complex to report to end users and does not provide an overall total score. We therefore also considered 2 simpler alternative models. In the bifactor model, all items load onto a general well-being factor, and each item also loads onto its specified subfactors. Subscale scores in the bifactor model reflect users’ scores on these subfactors controlling for overall well-being (eg, scores on the *Happiness* subscale reflect whether a user is more or less happy than would be expected, given their overall score). As such, subscale scores from the bifactor model may be more difficult for users to interpret. In the second-order model, the 7 subscales load onto an overall general factor, and the subscales are assumed to be uncorrelated once the common effect of this general is taken into account. The second-order model is a special case of the bifactor model, with proportionality constraints on particular weights [43]. However, this model corresponded to our common-sense idea of how the Unmind Index is structured (eg, the various happiness items reflect different facets of the *Happiness* subscale, and our various subscales reflect different facets of MHWB).

Model fit was evaluated using several indices: comparative fit index (CFI), Tucker-Lewis index (TLI), root mean square error of approximation (RMSEA), and standardized root mean residual (SRMR). The CFI and TLI measure whether a given model fits the data better than a more restricted baseline model, with the TLI applying a penalty to more complex models (and thus being the conservative index of the two). RMSEA is an absolute fit index, in that it assesses how far a hypothesized model is from a perfect model. SRMR outputs the average discrepancy between the model-estimated statistics and observed sample statistics. A model fit >.90 was considered acceptable for both CFI and TLI, and >.95 was considered good. For RMSEA and SRMR, a value between .06 and .08 was considered an acceptable fit, while a value <.06 was considered a good fit [44,45].

Given the large sample size, even extremely small differences in model fit are likely to be statistically significant. As a result, null hypothesis significance testing was not appropriate here, and we instead used information criteria (IC) for formal model comparison. The Akaike information criterion (AIC) is an estimate of expected out-of-sample prediction error, and the model with the lowest AIC is expected to provide the most accurate predictions on new data. The Bayesian information criterion (BIC) is proportional to an approximation of marginal likelihood of a model, and the model with the lowest BIC has the greatest posterior probability of being the true model, assuming one of the models considered is true. With large sample sizes, AIC will favor more complicated models than BIC, since an overcomplex model can still produce accurate predictions, given adequate data [46]. We therefore relied on the BIC when the criteria disagreed. Absolute IC values are not informative, so to facilitate comparisons between models, it is customary to subtract the score of the best fitting model from all models and report differences between the best model (ΔIC=0) and the competitors (ΔIC>0) [46].

#### Statistical Analysis: Test-Retest Reliability

One-week test-retest reliability for the Unmind Index was assessed by computing 2-way consistency intraclass correlation coefficients (ICC [C, 1]) using data collected from a subsample of the Study 2 population (n=238, after 12 dropouts). The sample size was based on a previously recommended item-respondent ratio of at least 1:5 [47].

#### Statistical Analysis: Internal Consistency

To determine the internal consistency of the Unmind Index, we computed the Cronbach α [48] given it is the most widely used index of the reliability of a scale to date. As the tau equivalence assumption of α is rarely met in practice [49], we also calculated coefficient omega (ω) [50] as an indicator of internal consistency. We found little difference between α and ω for each subscale.

#### Statistical Analysis: Convergent and Discriminant Validity

The existing measures of mental health and personality used in this study, and the Unmind Index subscales they were expected to correlate with, are summarized in Table 1. We expected the following to be negatively correlated: PHQ-9 [16] with the *Happiness* subscale, GAD-7 [17] with the *Calmness* subscale, the Hospital Anxiety and Depression Scale (HADS) [36] anxiety subscale with the *Calmness* subscale, HADS depression subscale with the *Happiness* subscale, the Perceived Stress Scale (PSS) [37] with the *Coping* subscale, and the ISI [18] with the *Sleep* subscale. We expected the following to be positively correlated: the physical health subscale of PROMIS-10 (Patient-Reported Outcomes Measurement Information System) Global Health [39] with the *Health* subscale, BIT [21] with the *Fulfilment* subscale, and WEMWBS [20] with the Unmind Index overall score.

To establish the discriminant validity of the Unmind Index, we also included the Ten-Item Personality Inventory (TIPI) [40], a brief scale that measures individual differences in the “Big Five” personality traits (extraversion, agreeableness, conscientiousness, emotional stability, and openness to experiences). These personality subscales were expected to correlate only weakly with the Unmind Index subscales, as the Unmind Index is intended to capture states of mental health, rather than static traits.

Pearson correlations were computed between the battery of convergent and discriminant validity measures and Unmind Index scores and adjusted for reliability (disattenuated) using the Cronbach α estimates for each measure:




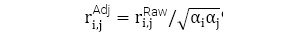




Given the strong associations typically found between various mental health measures [19], we assessed convergent validity by checking that the pattern of correlations of Unmind Index subscale scores with the relevant existing measures (eg, *Happiness* and PHQ-9) were (1) strong and (2) stronger than the correlation with less relevant existing measures (eg, *Happiness* and GAD-7). Discriminant validity was similarly assessed by checking that correlations between Unmind Index subscales and TIPI personality subscales were weak and weaker than correlations between the Unmind Index and mental health measures.

As an additional test of the validity of the Unmind Index, we explored the degree to which scores on the various Unmind Index subscales were predictive of participants’ self-reported health outcomes. These results are presented in Figure S4 in Multimedia Appendix 1.

#### Statistical Analysis: Measurement Invariance

It is important that the Unmind Index has the same factor structure (that is, measures the same constructs) and does not show bias across age and gender groups. To test this, we carried out measurement invariance analyses, fitting a series of additional second-order models where particular sets of parameters were allowed to vary between groups (multiple group CFA). Median participant age was 47 years, and so we classed participants as either older (>47 years, n=481), or younger (≤47 years, n=495); 475 participants identified as female, and 500 participants identified as male. One participant responded “Other/Prefer not to say” on the gender question and so was excluded from this analysis.

Measurement invariance was tested as follows [51]. We began by fitting a *configural invariance* model, where both groups have the same factor structure but all parameter values are allowed to differ between groups. If this model achieves a good fit, we can conclude that both groups show the same overall factor structure. We then compared this model to a *weak/metric invariance* model, where first- and second-level factor loadings are constrained to be equal across groups. If this constraint does not appreciably reduce model fit, we can conclude that factor weights are the same across groups. We then fit a *strong/scalar invariance* model, where item intercepts are also constrained to be equal, but factor means are allowed to differ between groups. If this does not show a poorer fit than the weak invariance model, we can conclude that item intercepts are equivalent across groups or, in other words, that any differences in factor scores are not driven by group differences on just some items. It is only appropriate to compare factor scores across groups if this final condition is met. We considered a constrained model to show poorer fit than the unconstrained alternative if the CFI decreased by more than 0.01 points [52] or if the BIC was lower for the unconstrained model. For completeness, we also report the SRMR, RMSEA, and TLI for each model.

#### Statistical Analysis: Group Differences

After establishing gender and age measurement invariance, we proceeded to explore gender and age differences in Unmind Index scores. To assess these trends statistically, we fit a linear regression model to each scale, with gender and age as predictors. These analyses were conducted on z-transformed scores, with an overall mean of 0 and standard deviation of 1. The regression weight for gender reflects the standardized difference between groups. The age predictor was divided by 10, so that the weight for age reflected the expected standardized difference between participants 10 years apart.

### Results

#### Factor Structure

Average inter-item correlation was examined, and no item displayed an average inter-item correlation above .8. Further, all items had an acceptable minimum average inter-item correlation (*r*>.2). No Heywood cases [53] were present.

**Figure 2:**
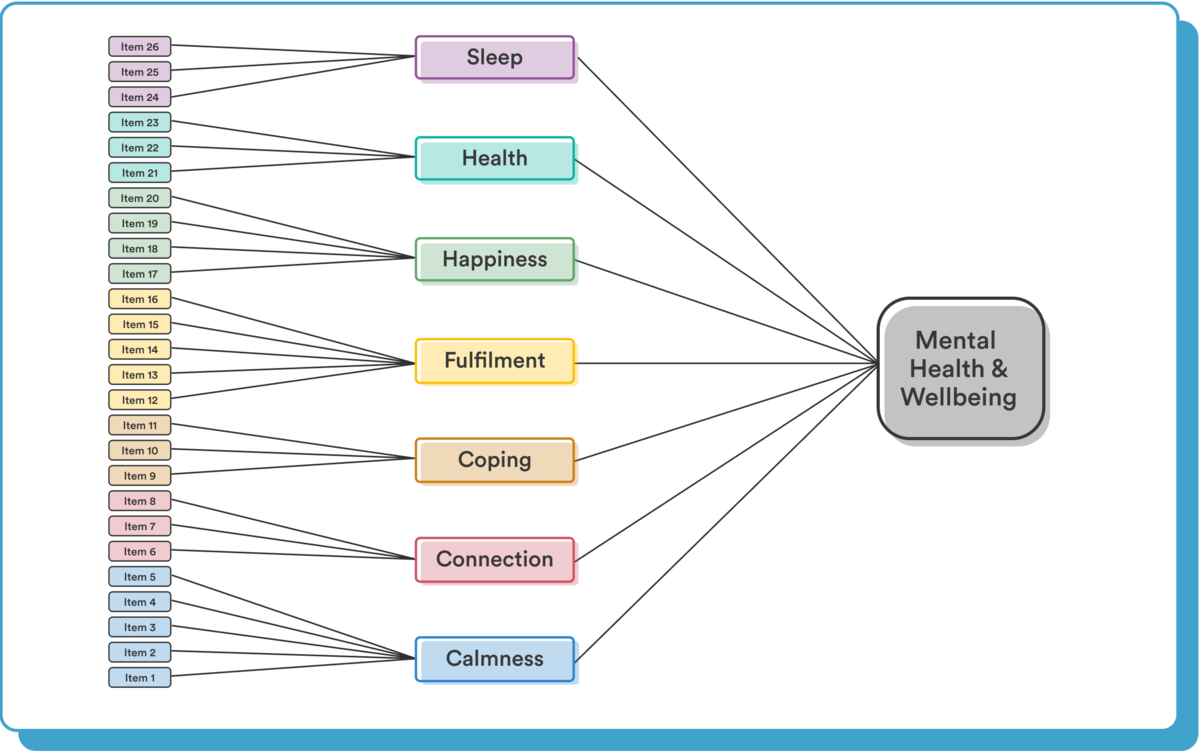
The second-order factor structure used for the Unmind Index.

CFA model comparison results are shown in Table 2. Parameter estimates for all models are reported in Tables S4-S8 in Multimedia Appendix 1. The correlated factors model provided a good fit to the data (SRMR=0.034, RMSEA=0.048, CFI=0.967, TLI=0.962), and was the superior model according to all model fit metrics considered. However, we considered this factor structure to be too complex to be interpretable by users. This structure also does not provide an overall MHWB score, one of our goals for the Unmind Index. We therefore decided not to use this model to score the Unmind Index. The bifactor and second-order models both provided good fits to the data. Although the bifactor model (SRMR=0.046, RMSEA=0.059, CFI=0.951, TLI=0.942, ΔAIC=306, ΔBIC=331) provided a slightly better fit than the second-order model (SRMR=0.049, RMSEA=0.062, CFI=0.943, TLI=0.936, ΔAIC=448, ΔBIC=380), the differences across fit indices were marginal. We therefore preferred the simpler second-order model to score the Unmind Index, as this model better accorded with our conceptualization of the Unmind Index and provided more easily interpretable factor scores. The second-order model is illustrated in Figure 2, and parameter estimates for this model are shown in Tables 3 and 4.

**Table 2:** Confirmatory factor analysis (CFA) model comparison results.

Model	LL^a^	χ^2^	K^b^	df^c^	SRMR^d^	RMSEA^e^	CFI^f^	TLI^g^	ΔAIC^h^	ΔBIC^i^
Correlated factors	–37047	807	73	278	.034	.048	.967	.962	0	0
Bifactor	–37196	1070	78	273	.046	.059	.951	.942	306	331
Second Order	–37285	1209	59	292	.049	.062	.943	.936	448	380

^a^LL: log-likelihood.

^b^K: number of parameters.

^c^df: degrees of freedom.

^d^SRMR: standardized root mean square residual.

^e^RMSEA: root mean square error of approximation.

^f^CFI: comparative fit index.

^g^TLI: Tucker-Lewis index.

^h^ΔAIC: difference in the Akaike information criteria between the model and the best-fitting model.

^i^ΔBIC: difference in the Bayesian information criteria between the model and the best-fitting model.

**Table 3:** Standardized factor loadings and residual item variances for the Unmind Index.

Factor and items			Factor loading (SE)		Residual variance (SE)		h^2a^
**Calmness**							
	Found it hard to stop (or control) worrying	.87 (.01)		.24 (.02)		.76	
	Had difficulty switching off	.76 (.02)		.42 (.03)		.58	
	Noticed that my body has been tense	.73 (.02)		.46 (.03)		.54	
	Worried that bad things might happen to me or others close to me	.67 (.02)		.56 (.03)		.44	
**Coping**							
	Felt confident that I can handle problems that come my way	.86 (.02)		.26 (.03)		.74	
	Been able to proactively manage my stress day to day	.74 (.02)		.45 (.03)		.55	
	Felt able to cope if something unexpected happens	.77 (.02)		.41 (.03)		.59	
**Health**							
	Felt like I am in a good state of health	.89 (.01)		.20 (.02)		.80	
	Been managing my health well	.88 (.01)		.23 (.02)		.77	
	Felt that my physical health is not as good as I'd like it to be (given my age/life circumstances)	.62 (.03)		.61 (.03)		.39	
**Sleep**							
	Slept well, all things considered (eg, such as caring for young children at night, snoring partner, shift work)	.90 (.01)		.19 (.02)		.81	
	Felt satisfied with my sleep	.91 (.01)		.18 (.02)		.82	
	Had trouble falling or staying asleep or waking up too early	.78 (.02)		.40 (.03)		.60	
**Fulfilment**							
	Felt a sense of accomplishment	.80 (.02)		.36 (.02)		.64	
	Felt that I am growing positively as a person	.77 (.02)		.41 (.03)		.59	
	Felt like I am leading a fulfilling life	.83 (.01)		.31 (.02)		.69	
	Been feeling good about myself as a person	.89 (.01)		.20 (.01)		.80	
	Been feeling cheerful or bright in my mood	.84 (.01)		.30 (.02)		.70	
**Connection**							
	Felt connected to people around me	.84 (.01)		.29 (.02)		.71	
	Felt like I have warm and trusting relationships with others	.84 (.01)		.30 (.03)		.70	
	Felt appreciated by others	.83 (.02)		.32 (.03)		.68	
**Happiness**							
	Had little interest in people or activities that I used to enjoy	.74 (.02)		.46 (.03)		.54	
	Been feeling down or sad in my mood	.86 (.01)		.25 (.02)		.75	
	Found it hard to motivate myself to engage with everyday tasks	.73 (.02)		.47 (.03)		.53	
	Felt disappointed in myself	.80 (.02)		.37 (.02)		.63	
	Tended to get stuck in a cycle of negativity in my head	.85 (.01)		.28 (.02)		.72	

^a^h^2^: item communality.

**Table 4:** Raw factor means, SDs, and standardized loadings onto the overall second-order factor.

Factor	Mean (SD)	Second-order factor loading (SE)
Calmness	2.92 (1.33)	.84 (.02)
Coping	2.85 (1.35)	.91 (.01)
Health	2.99 (1.18)	.79 (.02)
Sleep	2.56 (1.48)	.64 (.03)
Fulfilment	2.66 (1.31)	.94 (.01)
Connection	2.61 (1.19)	.76 (.02)
Happiness	3.03 (1.24)	.93 (.01)

#### Reliability and Consistency

All subscales showed excellent internal consistency, assessed by estimating Cronbach α and coefficient ω from the second-order CFA model: *Happiness*, α=.90, ω=.90; *Sleep*, α=.89, ω=.89; *Coping*, α=.83, ω=.83; *Calmness*, α=.84, ω=.85; *Health*, α=.83, ω=.83; *Connection*, α=.87, ω=.87; *Fulfilment*, α=.92, ω=.91. Internal consistency for the overall MHWB factor was also excellent: ω_H_ (McDonald hierarchical omega)=.92.

All subscales had excellent test-retest reliability after 1 week, based on ICCs using a 2-way mixed effects model; ICC(C, 1) scores (95% CI) for each subscale (Table 5) were as follows: *Happiness*, .84 (.79-.87); *Sleep*, .81 (.76-.85); *Coping*, .78 (.73-.83); *Calmness*, .85 (.81-.88); *Health*, .81 (.76-.85); *Connection*, .79 (.74-.83); *Fulfilment*, .85 (.81-.88); *Well-being*, .90 (.88-.92).

**Table 5:** Factor reliability estimates, based on internal consistency (Cronbach α and McDonald ω) and test-retest reliability (2-way consistency).

Factor	Internal consistency		Test-retest, ICC^a^ (C, 1)
	Cronbach α	McDonald ω	
Total score	-^b^	.92	.90
Happiness	.90	.90	.84
Sleep	.89	.89	.81
Coping	.83	.83	.78
Calmness	.84	.85	.85
Health	.83	.83	.81
Connection	.87	.87	.79
Fulfilment	.92	.91	.85

^a^ICC: intraclass correlation coefficient.

^b^Not applicable for second-order factors.

#### Convergent and Discriminant Validity

**Figure 3:**
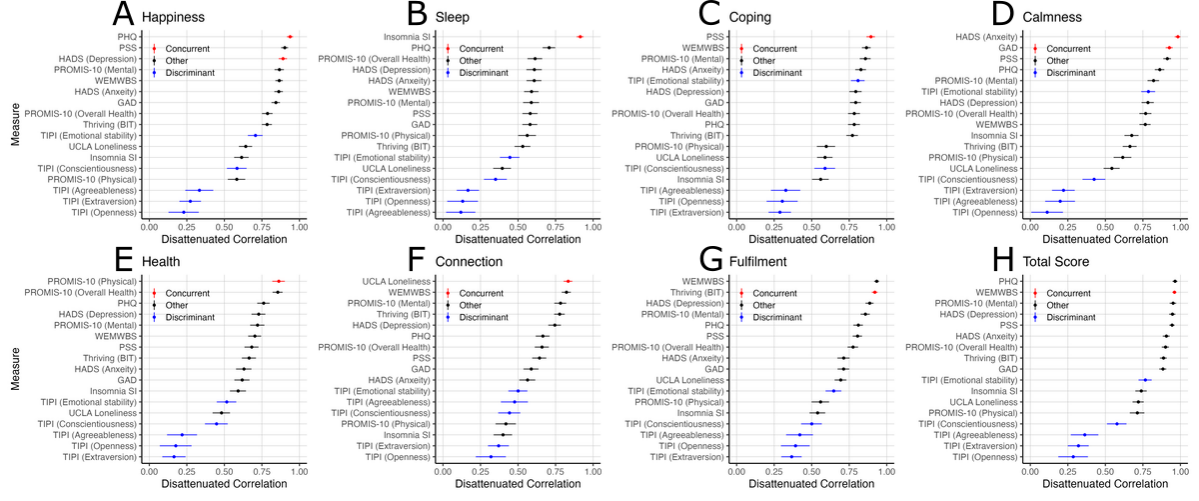
Disattenuated Pearson correlation coefficients between external measures of mental health and personality and the following Unmind Index subscales or total score: (A) Happiness, (B) Sleep, (C) Coping, (D) Calmness, (E) Health, (F) Connection, (G) Fulfilment, (H) Total Well-being score. BIT: Brief Inventory of Thriving; GAD: General Anxiety Disorder; HADS: Hospital Anxiety and Depression Scale; PHQ: Patient Health Questionnaire; PROMIS: Patient-Reported Outcomes Measurement Information System; PSS: Perceived Stress Scale; SI: Severity Index; TIPI: Ten-Item Personality Inventory; UCLA: University of California Los Angeles; WEMWBS: Warwick-Edinburgh Mental Well-being Scale.

**Figure 4:**
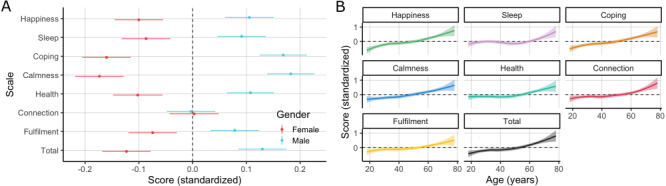
Standardized Unmind Index scores by (A) gender (mean and standard error of measurement within each group) and (B) age (LOWESS fit and standard error).

Correlations between Unmind Index subscales and external measures, with correction for attenuation, are shown in Figure 3. For clarity, correlation coefficients are reversed for relationships expected to be negative, so that positive correlations indicate relationships in the expected direction. Complete correlation tables and results without disattenuation are reported in Tables S1-S2 in Multimedia Appendix 1. It is well-established that mental health measures intended to measure a variety of conditions tend to correlate strongly with each other [19]. Unmind Index subscale scores were also strongly intercorrelated (Table 6). As a result, most Unmind Index subscales correlated strongly with a range of external measures (Figure 4). Importantly, however, correlations between subscales and external measures intended to reflect similar constructs were very strong and, in almost all cases, stronger than those between subscales and the remaining external mental health measures, demonstrating convergent validity.

**Table 6:** Observed correlations between Unmind Index scales.

Variable	Calmness	Coping	Health	Sleep	Fulfilment	Connection	Happiness	Total
Calmness	-^a^	0.67 (0.02)^b^	0.55 (0.03)	0.56 (0.03)	0.60 (0.03)	0.45 (0.03)	0.79 (0.02)	0.83 (0.02)
Coping	0.67 (0.02)	-	0.57 (0.03)	0.48 (0.03)	0.75 (0.02)	0.59 (0.03)	0.72 (0.02)	0.84 (0.02)
Health	0.55 (0.03)	0.57 (0.03)	-	0.49 (0.03)	0.63 (0.02)	0.45 (0.03)	0.61 (0.03)	0.75 (0.02)
Sleep	0.56 (0.03)	0.48 (0.03)	0.49 (0.03)	-	0.52 (0.03)	0.38 (0.03)	0.52 (0.03)	0.69 (0.02)
Fulfilment	0.60 (0.03)	0.75 (0.02)	0.63 (0.02)	0.52 (0.03)	-	0.72 (0.02)	0.77 (0.02)	0.89 (0.01)
Connection	0.45 (0.03)	0.59 (0.03)	0.45 (0.03)	0.38 (0.03)	0.72 (0.02)	-	0.59 (0.03)	0.73 (0.02)
Happiness	0.79 (0.02)	0.72 (0.02)	0.61 (0.03)	0.52 (0.03)	0.77 (0.02)	0.59 (0.03)	-	0.91 (0.01)
Total	0.83 (0.02)	0.84 (0.02)	0.75 (0.02)	0.69 (0.02)	0.89 (0.01)	0.73 (0.02)	0.91 (0.01)	-

^a^Not applicable.

^b^Values in parentheses indicate standard error.

There were several moderate exceptions to this pattern. The Unmind Index *Happiness* subscale was strongly related to the PHQ-9 and HADS depression subscale, as expected, but was similarly related to the PSS stress measure. This suggests our *Happiness* subscale captures a broader construct than these clinical depression inventories do. This did not diminish the predicted association between the Unmind Index *Coping* subscale and the PSS. Although the Unmind Index *Fulfilment* subscale was strongly correlated with the BIT, as expected, its correlation with the WEMWBS well-being scale was slightly stronger. Finally, the Unmind Index total score was strongly associated with many measures, although this is unsurprising given that this scale is a composite of our 7 subscales, and was most strongly correlated with WEMWBS, as expected.

Correlations between Unmind Index subscales and 4 of the 5 TIPI personality subscales (extraversion, agreeableness, conscientiousness, and openness) were generally smaller than those between the Unmind Index and any mental health measures and close to 0 in some cases, demonstrating reasonable discriminant validity. However, the TIPI emotional stability subscale (“I see myself as anxious, easily upset” [reverse-coded] and “I see myself as calm, emotionally stable”) was moderately correlated with several of our subscales. It should be noted that the test-retest reliability of this TIPI subscale is estimated to be only .70 [40], suggesting that it may, in part, capture state rather than trait emotional stability.

#### Measurement Invariance

Gender measurement invariance results are shown in Table 7. The configural invariance model achieved good model fit across all indices. Adding metric and scalar constraints led to extremely small changes in fit and improvements in BIC, indicating that scalar invariance held across gender groups; therefore, Unmind Index scores can be directly compared between male and female users.

**Table 7:** Measurement invariance by gender.

Invariance model	Constraints	df^a^	χ^2^	CFI^b^	BIC^c^	SRMR^d^	RMSEA^e^	TLI^f^
Configural	Factor structure	584	1796	.936	235	.051	.065	.929
Weak/metric^g^	Structure and loadings	609 (+25)	1819 (+23)	.936 (–.000)	86 (–149)	.053 (+.002)	.064 (–.001)	.932 (+.003)
Strong/scalar^g^	Structure, loadings, and item intercepts	627 (+18)	1857 (+38)	.935 (–.001)	0 (–86)	.054 (+.001)	.063 (–.000)	.933 (+.001)

^a^df: degrees of freedom.

^b^CFI: comparative fit index.

^c^BIC: Bayesian information criterion.

^d^SRMR: standardized root mean square residual.

^e^RMSEA: root mean square error of approximation.

^f^TLI: Tucker-Lewis index.

^g^Values in parentheses provide the comparisons with the less-constrained models reported in the previous row, shown as the difference between the values.

Age measurement invariance results are shown in Table 8 and reveal similar findings, indicating that scalar invariance holds across age groups; therefore, Unmind Index scores can be directly compared between older and younger users.

**Table 8:** Measurement invariance by age group (≥48 years vs ≤47 years).

Invariance model	Constraints	df^a^	χ^2^	CFI^b^	BIC^c^	SRMR^d^	RMSEA^e^	TLI^f^
Configural	Factor structure	584	1728	.939	147	.051	.063	.932
Weak/metric^g^	Structure and loadings	609 (+25)	1778 (+50)	.937 (–.001)	25 (–122)	.059 (+.008)	.063 (–.001)	.933 (+.001)
Strong/scalar^g^	Structure, loadings, and item intercepts	627 (+18)	1877 (+99)	.933 (–.004)	0 (–25)	.060 (+.000)	.064 (+.001)	.931 (–.003)

^a^df: degrees of freedom.

^b^CFI: comparative fit index.

^c^BIC: Bayesian information criterion.

^d^SRMR: standardized root mean square residual.

^e^RMSEA: root mean square error of approximation.

^f^TLI: Tucker-Lewis index.

^g^Values in parentheses provide the comparisons with the less-constrained models reported in the previous row, shown as the difference between the values.

#### Group Differences

Female participants scored significantly lower than males on all scales except for Connection: total score (95% CI), b=–0.26 (–0.38 to –0.14); *Happiness*, b=–0.22 (–0.34 to –0.10); *Calmness*, b = –0.37 (–0.49 to –0.25); *Coping*, b=–0.34 (–0.46 to –0.22); *Sleep*, b=–0.18 (–0.31 to –0.06); *Health*, b=–0.22 (–0.34 to –0.09); *Fulfilment*, b=–0.16 (–0.28 to –0.04); *Connection*, b=–0.00 (–0.13 to 0.12). Older participants scored significantly higher on all scales, although the effect on *Sleep* was somewhat smaller: total score, b=0.15 (0.12 to 0.19); *Happiness*, b=0.18 (0.14 to 0.22); *Calmness*, b=0.15 (0.11 to 0.19); *Coping*, b=0.17 (0.13 to 0.20); *Sleep*, b=0.06 (0.02 to 0.10); *Health*, b=0.10 (0.06 to 0.14); *Fulfilment*, b=0.10 (0.06 to 0.14); *Connection*, b=0.11 (0.07 to 0.15).

## Discussion

### Summary

In Study 1A, we reported the process by which candidate items for the Unmind Index were generated, screened for validity, and initially clustered into subdomains. In Study 1B, we used an iterative data-driven approach to shorten the list of candidate items, used multifactor EFA to identify the underlying factor structure of these items, and finally integrated this data-driven factor structure with practical and theoretical considerations to establish the items and factor structure of the Unmind Index. This consists of 26 items and 7 subscales: *Happiness*, capturing positive mood or the absence of depressive symptoms; *Coping*, capturing perceived capacity to deal with stress; *Health*, capturing physical health and its impact on everyday life; *Sleep*, capturing sleep quality and its impact on functioning; *Calmness*, capturing calm or the absence of anxiety symptoms; *Connection*, capturing a sense of feeling supported and valued; and *Fulfilment,* capturing a sense of accomplishment, growth, or purpose.

These subscales differ from the 7 factors we used to guide the item generation process: *Happiness*, *Coping*, *Health*, *Sleep*, *Calmness*, *Energy*, and *Vitality*. We found that items intended to measure *Energy* did not load onto a single factor, and so, this construct was eliminated. Items intended to measure *Vitality* formed 2 factors: *Connection*, capturing the social aspects of the vitality construct, and *Fulfilment*, capturing the self-directed aspects. Although the EFA results indicated that the *Calmness* factor could be partitioned into *Worry* and *Tension*, we chose to maintain the single factor for practical reasons.

In Study 2, we validated the Unmind Index with new participants. We established that a second-order factor structure provides good fit to the data, that the scales have good internal and test-retest reliability, and that the subscales correlate as expected with existing measures of MHWB and do not correlate strongly with personality scales, with the exception of the emotional stability trait. Finally, the Unmind Index displayed measurement invariance with regard to gender and age, meaning that scores can be validly compared across these groups.

Although the second-order factor model fit the data well, it was outperformed by the correlated factors model, which directly modeled the correlations between all 7 subscales. This implies that some subscales are more closely related than others, a result that is confirmed by the information presented in Table 5. This is consistent with a growing body of work showing that the symptoms of many mental health issues largely overlap [19,54], suggesting that a smaller number of transdiagnostic features, such as cognitive inflexibility or repetitive negative thinking may underpin many mental health problems [55]. In particular, the *Calmness* and *Happiness* subscales were strongly correlated. This is unsurprising, given that these subscales are negatively associated with existing measures of anxiety and depression, respectively, and that anxiety and depression are strongly linked [56]. However, although the second-order model did not utilize this information, it provided a clear, practical structure for communicating results to users and is preferred for this reason.

### Scoring

It is important that scores on the Unmind Index are easy for users to understand, can be compared across subscales, and can be compared to a meaningful reference value. For this reason, Unmind Index subscale scores reported to users are standardized to population norms estimated from this validation study, with a mean of 100 and a standard deviation of 15. This makes scores directly interpretable by users in a way that is not the case for unstandardized measures and allows for direct comparisons between subscale scores. It is also in line with recent appeals [57] that mental health measures should be reported in a way that makes scores across measures comparable.

### Limitations and Future Directions

A number of limitations and directions for future work remain. The Unmind Index asks respondents to report their mental state over the previous 2 weeks. It is not yet known to what extent Unmind Index scores fluctuate over time, although our high test-retest reliability indicates that scores do not change considerably over a single week. Further work is also needed to determine to what degree the Unmind Index is sensitive to changes in mental health. To address this, we are currently including the Unmind Index as a secondary outcome measure in randomized controlled efficacy trials, with the intention of testing whether pre-post changes in existing measures such as the PHQ-8 are predictive of changes in Unmind Index scores.

We reported results from (exploratory and confirmatory) linear factor analyses in this paper. However, responses to the Unmind Index are given on a 6-point Likert scale, from “No days” to “Every day.” In future work, we will reanalyze these data using multivariate item response theory modelling [58]. Doing so will allow us to better understand how users make use of this response scale and may lead to an adaptive version of the Unmind Index, where the questions asked are calibrated to individual users’ score profiles.

Lastly, our validation is currently limited to a UK population, and we acknowledge that the subjective experience of mental health and conceptualization of well-being can vary across cultures [59]. We are planning future studies to validate the Unmind Index in other geographies and establish relevant norms and scoring bandings.

### Conclusion

This work demonstrated the Unmind Index is a robust measure of MHWB that is underpinned by a general factor and 7 underlying constructs. We suggest that MHWB can usefully be measured in conjunction, challenging the false dichotomy (and associated stigma) that is perpetuated when mental ill health and mental well-being are described and measured separately. This is particularly relevant for assessment offered to working adults who are likely to encompass the full spectrums of MHWB. We would encourage other mHealth app developers to capture the broader aspects of positive well-being when aiming to measure mental health.

## Appendix

Multimedia Appendix 1 Supplementary materials.

## Abbreviations

AIC: Akaike information criterion
BIC: Bayesian information criterion
BIT: Brief Inventory of Thriving
CFA: confirmatory factor analysis
CFI: comparative fit index
DSM: Diagnostic and Statistical Manual of Mental Disorders
EFA: exploratory factor analysis
GAD-7: General Anxiety Disorder 7
HADS: Hospital Anxiety and Depression Scale
I-CVI: item content validity index
IC: information criteria
ICC: intraclass correlation coefficient
ICD: International Classification of Diseases
ISI: Insomnia Severity Index
mHealth: mobile health.
MHWB: mental health and well-being
PHQ-9: Patient Health Questionnaire 9
PROMIS: Patient-Reported Outcomes Measurement Information System
PSS: Perceived Stress Scale
RMSEA: root mean square error of approximation
SRMR: standardized root mean residual
TIPI: Ten-Item Personality Inventory
TLI: Tucker-Lewis index
WEMWBS: Warwick-Edinburgh Mental Well-being Scale
