# Supplementary material for: A New Digital Assessment of Mental Health and Well-being in the Workplace: Development and Validation of the Unmind Index
Source: JMIR Ment Health. 2022 Jan 17;9(1):e34103. doi: 10.2196/34103 (PMC8804960; doi:10.2196/34103)
Supplement: Multimedia Appendix 1 [file mental_v9i1e34103_app1.docx]

Supplementary Materials

for

The Unmind Index: development and UK validation of a new digital assessment of mental health and wellbeing

­­

Anika Sierk, Eoin Travers, Marcos Economides,
Bao Sheng Loe, Luning Sun, Heather Bolton


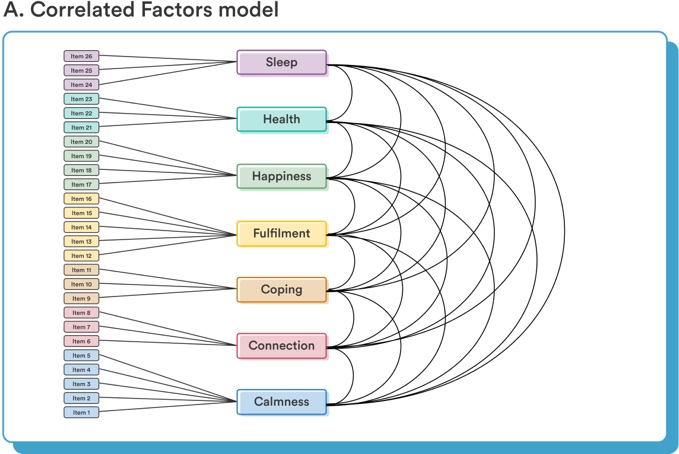

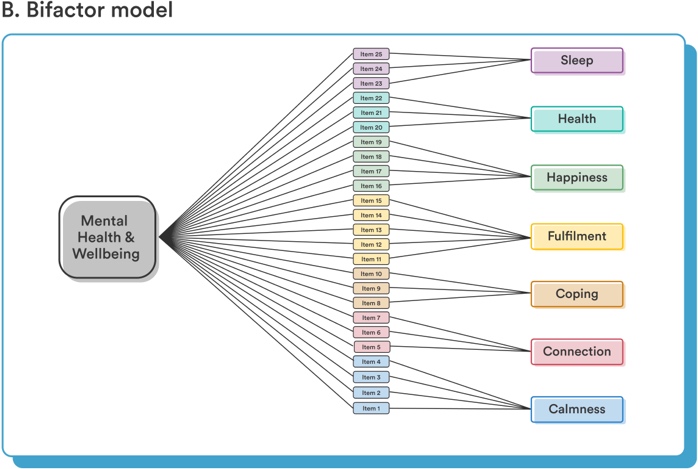


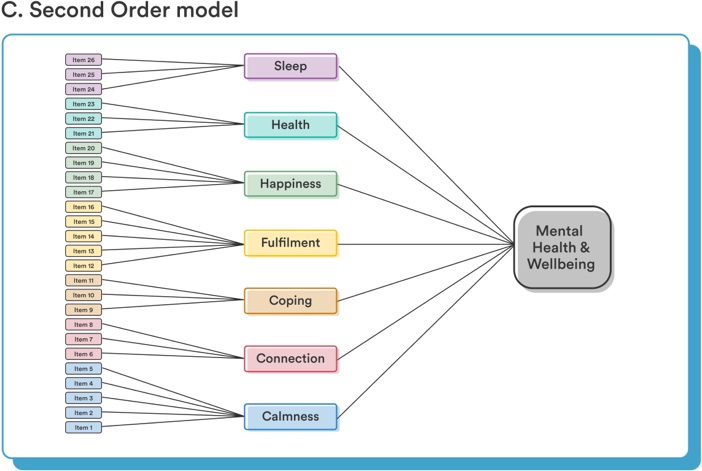


**Figure S1.** Confirmatory factor analysis model structures used in Study 2.

**Table S1.** Pearson correlation coefficients for correlations between Unmind Index subscales and total score and existing measures of mental health and wellbeing, and personality traits.

|  | Calmness | Coping | Health | Sleep | Fulfilment | Connection | Happiness | Total Score |
| --- | --- | --- | --- | --- | --- | --- | --- | --- |
| GAD | -.82 | -.70 | -.54 | -.53 | -.66 | -.53 | -.77 | -.81 |
| HADS (Anxiety) | +.85 | +.71 | +.54 | +.54 | +.65 | +.50 | +.77 | +.82 |
| PSS | -.80 | -.78 | -.59 | -.53 | -.74 | -.58 | -.82 | -.86 |
| PROMIS (Physical) | +.50 | +.48 | +.69 | +.46 | +.47 | +.34 | +.48 | +.60 |
| PROMIS (Mental) | +.69 | +.72 | +.60 | +.51 | +.76 | +.67 | +.76 | +.84 |
| PROMIS (Total) | +.67 | +.68 | +.74 | +.55 | +.70 | +.58 | +.71 | +.81 |
| ISI | -.59 | -.48 | -.51 | -.82 | -.49 | -.36 | -.55 | -.67 |
| BIT | +.59 | +.68 | +.58 | +.48 | +.85 | +.70 | +.72 | +.82 |
| Loneliness | -.49 | -.52 | -.43 | -.36 | -.65 | -.76 | -.59 | -.67 |
| PHQ | -.75 | -.68 | -.66 | -.63 | -.74 | -.59 | -.84 | -.87 |
| HADS (Depression) | +.67 | +.67 | +.62 | +.53 | +.79 | +.65 | +.78 | +.84 |
| WEMWBS | +.69 | +.77 | +.62 | +.54 | +.87 | +.75 | +.80 | +.89 |
| TIPI (Extraversion) | +.18 | +.23 | +.13 | +.14 | +.31 | +.30 | +.23 | +.27 |
| TIPI (Agreeableness) | +.12 | +.20 | +.13 | +.08 | +.27 | +.30 | +.21 | +.23 |
| TIPI (Conscient.) | +.32 | +.43 | +.33 | +.27 | +.39 | +.34 | +.45 | +.45 |
| TIPI (Emo. Stability) | +.64 | +.65 | +.41 | +.37 | +.54 | +.41 | +.59 | +.64 |
| TIPI (Openness) | +.07 | +.18 | +.10 | +.08 | +.24 | +.19 | +.14 | +.18 |

**Table S2.** Disattenuated Pearson correlation coefficients for correlations between Unmind Index subscales and total score and existing measures of mental health and wellbeing, and personality traits. Cronbach’s alpha reliability estimates were used to disattenute the estimated correlations: $r_{i,j}^{\text{Adj}}=r_{i,j}^{\text{Raw}}/\sqrt{\alpha_{i}\alpha_{j}}$

|  | Calmness | Coping | Health | Sleep | Fulfilment | Connection | Happiness | Total Score |
| --- | --- | --- | --- | --- | --- | --- | --- | --- |
| GAD | -.93 | -.79 | -.62 | -.58 | -.71 | -.59 | -.84 | -.88 |
| HADS (Anxiety) | +.98 | +.83 | +.63 | +.61 | +.71 | +.56 | +.86 | +.91 |
| PSS | -.91 | -.89 | -.68 | -.58 | -.81 | -.64 | -.90 | -.94 |
| PROMIS (Physical) | +.62 | +.60 | +.86 | +.56 | +.56 | +.42 | +.58 | +.71 |
| PROMIS (Mental) | +.82 | +.86 | +.72 | +.59 | +.86 | +.78 | +.87 | +.95 |
| PROMIS (Total) | +.77 | +.78 | +.86 | +.61 | +.78 | +.66 | +.79 | +.90 |
| ISI | -.68 | -.56 | -.59 | -.91 | -.54 | -.4 | -.61 | -.74 |
| BIT | +.66 | +.77 | +.67 | +.53 | +.92 | +.78 | +.78 | +.89 |
| Loneliness | -.54 | -.59 | -.48 | -.39 | -.69 | -.83 | -.64 | -.72 |
| PHQ | -.86 | -.78 | -.76 | -.71 | -.81 | -.67 | -.94 | -.96 |
| HADS (Depression) | +.78 | +.79 | +.73 | +.61 | +.89 | +.75 | +.89 | +.95 |
| WEMWBS | +.77 | +.87 | +.70 | +.59 | +.93 | +.82 | +.87 | +.96 |
| TIPI (Extraversion) | +.22 | +.29 | +.16 | +.17 | +.37 | +.37 | +.27 | +.32 |
| TIPI (Agreeableness) | +.20 | +.33 | +.22 | +.12 | +.42 | +.48 | +.33 | +.36 |
| TIPI (Conscient.) | +.43 | +.59 | +.45 | +.35 | +.50 | +.44 | +.58 | +.58 |
| TIPI (Emo. Stability) | +.79 | +.81 | +.52 | +.45 | +.65 | +.50 | +.71 | +.77 |
| TIPI (Openness) | +.11 | +.30 | +.18 | +.13 | +.39 | +.32 | +.23 | +.29 |


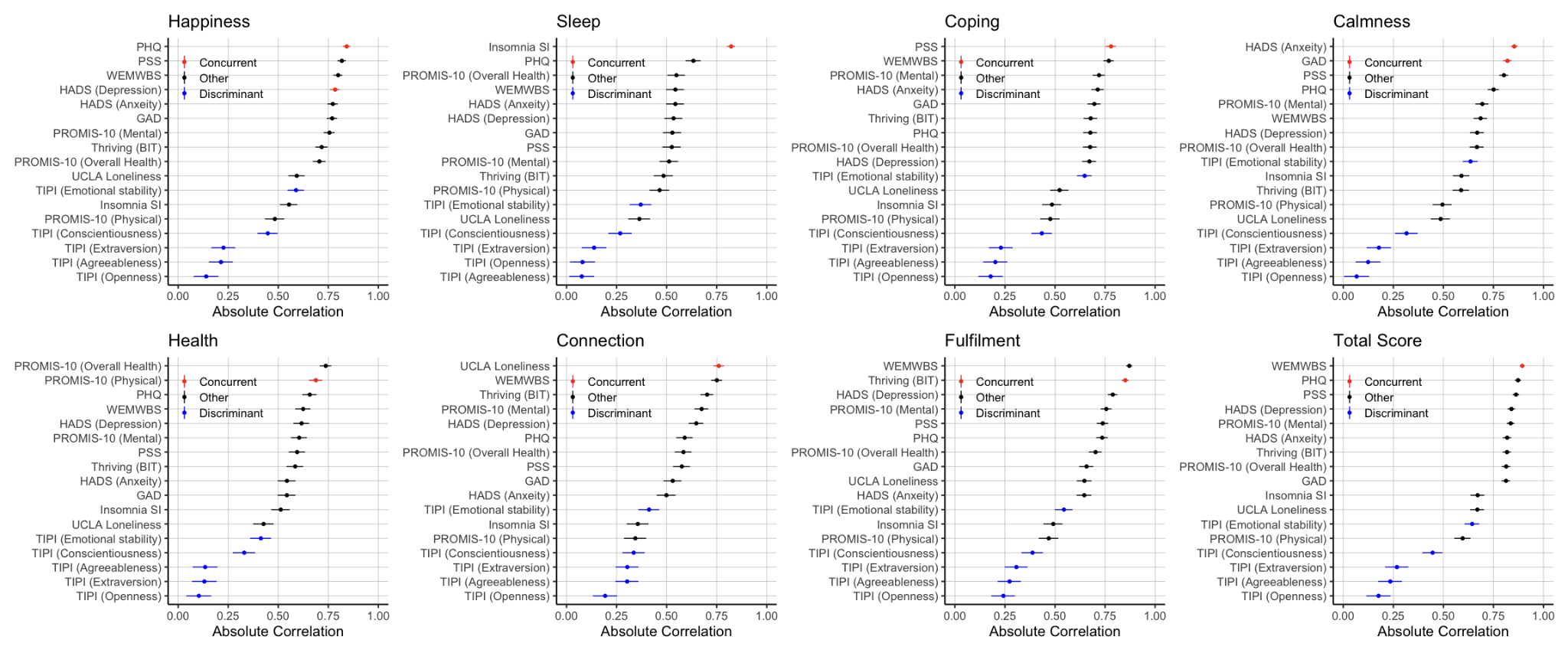
**Figure S2.** Pearson correlation coefficients between Index subscales (or the overall Wellbeing scale) and external measures of mental health and personality. Values in red show wellbeing state measures expected to correlate strongly with the Index subscale (concurrent validity). Values in blue show personality trait measures, expected to correlate weakly (discriminant validity).

## Concurrent Validity

As an additional test of the validity of the Unmind Index, we examined the relationship between participants’ Unmind Index scores and their responses to a set of self-report health questions. Participants were asked if they were currently taking any medication for physical or mental health issues, the number of times they had visited their GP (doctor) in the last six months, the number sick days they had taken off work in the last six months, and to rate their overall health on a scale from 0 to 10. Distributions of responses to these questions are shown in Figure S2.

For each question, and for each of the eight Unmind Index scores (total score, plus seven subscales), we fit a generalised linear model (GLM) with the self-reported health outcome as the outcome, the Unmind Index score as a predictor, and age and gender as covariates. One participant who responded “Other/Prefer not to say” on the gender question was excluded from these analyses. Controlling for age in particular is necessary because older participants tended to have higher Unmind Index scores, but poorer self-reported health outcomes. We used binomial logistic regression to model the yes/no medication questions (any medicine, medicine for mental health, and medicine for physical health), and ordinal logistic regression for the number of GP visits, number of sick days (excluding participants who selected the N/A option), and overall health rating. Unmind Index scores were z-scored prior to model-fitting so that regression weights can be compared across the various scores.


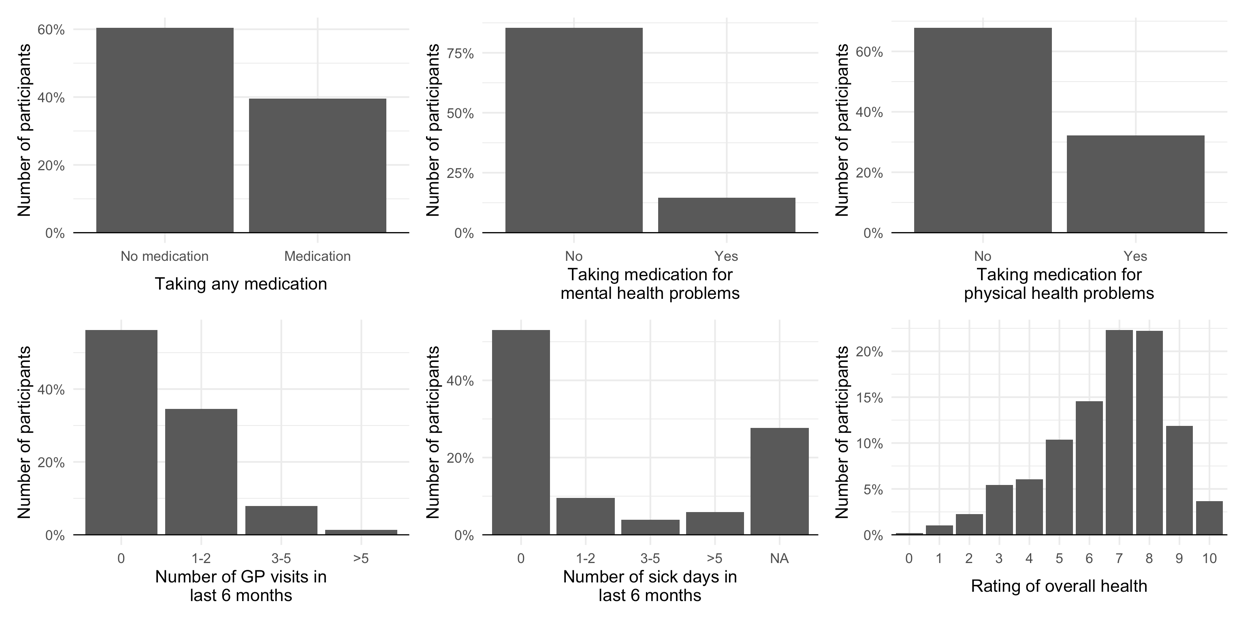


**Figure S3.** Distribution of responses for self-report health outcomes.

Regression weights for each Unmind Index score, predicting each self-report health outcome, are shown in Figure S3. All scores were negative predictors of medication use, although the effect for the *Connection* subscale was weaker for both mental health and physical health problems, and non-significant for physical health problems. The *Health* subscale was the strongest predictor of medication use for physical health problems, while the *Coping* was the strongest predictor of medication use for mental health problems, albeit by a small margin. Similar results were found for the number of GP visits and the number of sick days taken, with the *Health* subscale being the strongest predictor by a small margin, and the *Connection* subscale being the weakest predictor and not statistically significant. All subscales were significant predictors overall health ratings, and the association with the *Health* subscale was approximately twice as strong as the association with any other subscale. These results show that the Unmind Index is a robust predictor of self-reported health outcomes, that the *Health* subscale is the strongest predictor of these outcomes, and that these associations reflect nuances between scales, for instance that the *Coping* subscale is the strongest predictor of mental health medication use.


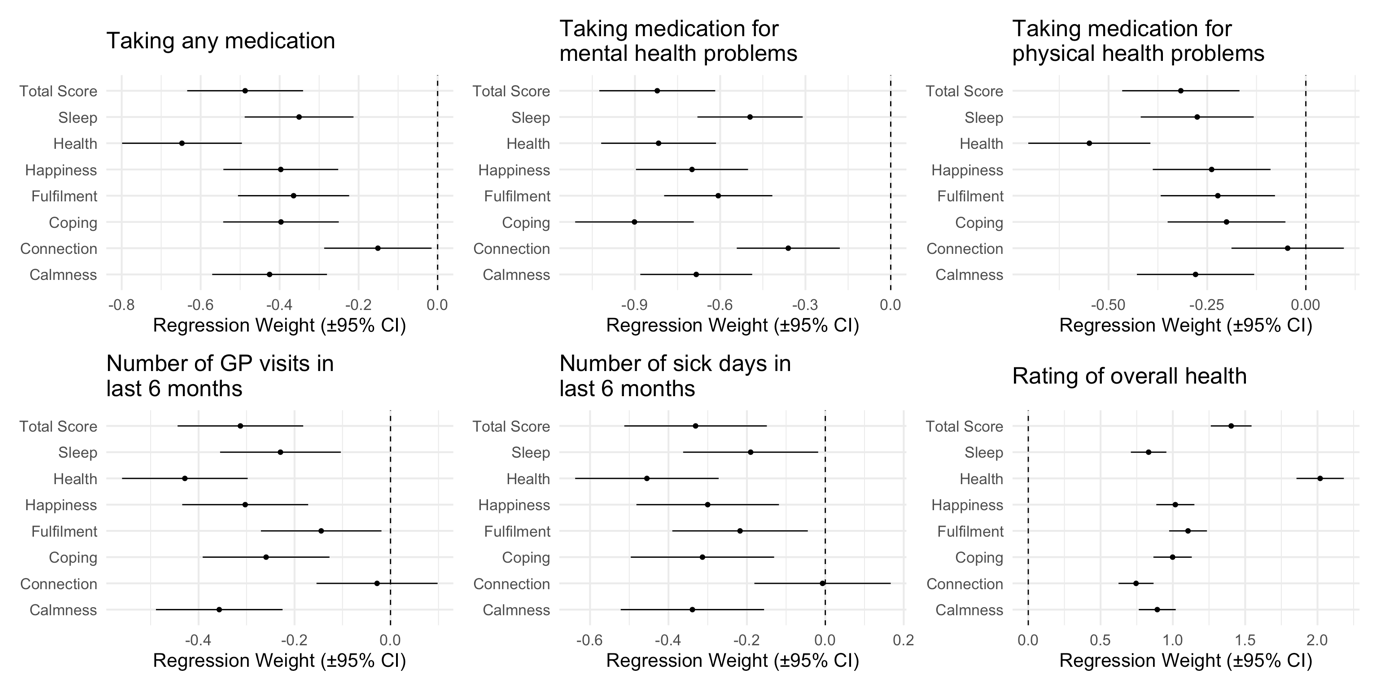


**Figure S4.** Concurrent validity of Unmind Index scores as predictors of self-report health outcomes. For each outcome and Unmind Index subscale, we fit a GLM with the subscale score as a predictor, and age and gender as covariates. Values show standardised regression weights β.

## Item Order

**Table S3.** When the Unmind Index is presented with a fixed item order, as is the case on the Unmind platform, the order shown below should be used.

| Item | Item text | Subscale | Reverse scored |
| --- | --- | --- | --- |
| 1 | been feeling cheerful or bright in my mood | Fulfilment | No |
| 2 | felt like I am in a good state of health | Health | No |
| 3 | felt appreciated by others | Connection | No |
| 4 | felt disappointed in myself | Happiness | Yes |
| 5 | felt confident that I can handle problems that come my way | Coping | No |
| 6 | slept well, all things considered (e.g. such as caring for young children at night, snoring partner, shift work, etc) | Sleep | No |
| 7 | worried that bad things might happen to me or others close to me | Calmness | Yes |
| 8 | felt like I have warm and trusting relationships with others | Connection | No |
| 9 | felt able to cope if something unexpected happens | Coping | No |
| 10 | had little interest in people or activities that I used to enjoy | Happiness | Yes |
| 11 | been able to proactively manage my stress day to day | Coping | No |
| 12 | been feeling down or sad in my mood | Happiness | Yes |
| 13 | found it hard to motivate myself to engage with everyday tasks | Happiness | Yes |
| 14 | been feeling good about myself as a person | Fulfilment | No |
| 15 | felt satisfied with my sleep | Sleep | No |
| 16 | felt like I am leading a fulfilling life | Fulfilment | No |
| 17 | tended to get stuck in a cycle of negativity in my head | Happiness | Yes |
| 18 | felt that I am growing positively as a person | Fulfilment | No |
| 19 | noticed that my body has been tense | Calmness | Yes |
| 20 | had difficulty switching off | Calmness | Yes |
| 21 | had trouble falling or staying asleep, or waking up too early | Sleep | Yes |
| 22 | felt that my physical health is not as good as I'd like it to be (given my age/life circumstances) | Health | Yes |
| 23 | felt a sense of accomplishment | Fulfilment | No |
| 24 | found it hard to stop (or control) worrying | Calmness | Yes |
| 25 | been managing my health well | Health | No |
| 26 | felt connected to people around me | Connection | No |

**Table S4.** Standardised factor loadings and residual item variances (± standard errors) for the second-order CFA model. *Note: h^2^ = Item communality.*

| Factor | Item | Factor  Loading | Residual Variance | h^2^ |
| --- | --- | --- | --- | --- |
| Calmness | found it hard to stop (or control) worrying | .87 (±.01) | .24 (±.02) | .76 |
| Calmness | had difficulty switching off | .76 (±.02) | .42 (±.03) | .58 |
| Calmness | noticed that my body has been tense | .73 (±.02) | .46 (±.03) | .54 |
| Calmness | worried that bad things might happen to me or others close to me | .67 (±.02) | .56 (±.03) | .44 |
| Coping | felt confident that I can handle problems that come my way | .86 (±.02) | .26 (±.03) | .74 |
| Coping | been able to proactively manage my stress day to day | .74 (±.02) | .45 (±.03) | .55 |
| Coping | felt able to cope if something unexpected happens | .77 (±.02) | .41 (±.03) | .59 |
| Health | felt like I am in a good state of health | .89 (±.01) | .20 (±.02) | .80 |
| Health | been managing my health well | .88 (±.01) | .23 (±.02) | .77 |
| Health | felt that my physical health is not as good as I'd like it to be (given my age/life circumstances) | .62 (±.03) | .61 (±.03) | .39 |
| Sleep | slept well, all things considered (e.g. such as caring for young children at night, snoring partner, shift work, etc) | .90 (±.01) | .19 (±.02) | .81 |
| Sleep | felt satisfied with my sleep | .91 (±.01) | .18 (±.02) | .82 |
| Sleep | had trouble falling or staying asleep, or waking up too early | .78 (±.02) | .40 (±.03) | .60 |
| Fulfilment | felt a sense of accomplishment | .80 (±.02) | .36 (±.02) | .64 |
| Fulfilment | felt that I am growing positively as a person | .77 (±.02) | .41 (±.03) | .59 |
| Fulfilment | felt like I am leading a fulfilling life | .83 (±.01) | .31 (±.02) | .69 |
| Fulfilment | been feeling good about myself as a person | .89 (±.01) | .20 (±.01) | .80 |
| Fulfilment | been feeling cheerful or bright in my mood | .84 (±.01) | .30 (±.02) | .70 |
| Connection | felt connected to people around me | .84 (±.01) | .29 (±.02) | .71 |
| Connection | felt like I have warm and trusting relationships with others | .84 (±.01) | .30 (±.03) | .70 |
| Connection | felt appreciated by others | .83 (±.02) | .32 (±.03) | .68 |
| Happiness | had little interest in people or activities that I used to enjoy | .74 (±.02) | .46 (±.03) | .54 |
| Happiness | been feeling down or sad in my mood | .86 (±.01) | .25 (±.02) | .75 |
| Happiness | found it hard to motivate myself to engage with everyday tasks | .73 (±.02) | .47 (±.03) | .53 |
| Happiness | felt disappointed in myself | .80 (±.02) | .37 (±.02) | .63 |
| Happiness | tended to get stuck in a cycle of negativity in my head | .85 (±.01) | .28 (±.02) | .72 |

**Table S5.** Raw factor means, standard deviations, and standardised loadings onto to the overall second-order factor for the second-order CFA model (± standard errors).

| Factor | Mean | SD | Second-order Factor Loading |
| --- | --- | --- | --- |
| Calmness | 2.92 | 1.33 | .84 (±.02) |
| Coping | 2.85 | 1.35 | .91 (±.01) |
| Health | 2.99 | 1.18 | .79 (±.02) |
| Sleep | 2.56 | 1.48 | .64 (±.03) |
| Fulfilment | 2.66 | 1.31 | .94 (±.01) |
| Connection | 2.61 | 1.19 | .76 (±.02) |
| Happiness | 3.03 | 1.24 | .93 (±.01) |

**Table S6.** Standardised factor loadings and residual item variances (± standard errors) for correlated factors CFA model. *Note: h^2^ = Item communality.*

| Factor | Item | Factor Loading | Residual Variance | Communality |
| --- | --- | --- | --- | --- |
| Calmness | found it hard to stop (or control) worrying | .86 (±.01) | .26 (±.02) | 0.74 |
| Calmness | had difficulty switching off | .77 (±.02) | .41 (±.03) | 0.59 |
| Calmness | noticed that my body has been tense | .74 (±.02) | .46 (±.03) | 0.54 |
| Calmness | worried that bad things might happen to me or others close to me | .66 (±.02) | .56 (±.03) | 0.44 |
| Coping | felt confident that I can handle problems that come my way | .86 (±.01) | .25 (±.03) | 0.75 |
| Coping | been able to proactively manage my stress day to day | .74 (±.02) | .46 (±.03) | 0.54 |
| Coping | felt able to cope if something unexpected happens | .77 (±.02) | .40 (±.03) | 0.60 |
| Health | felt like I am in a good state of health | .89 (±.01) | .20 (±.02) | 0.80 |
| Health | been managing my health well | .88 (±.01) | .23 (±.02) | 0.77 |
| Health | felt that my physical health is not as good as I'd like it to be (given my age/life circumstances) | .62 (±.03) | .62 (±.03) | 0.38 |
| Sleep | slept well, all things considered (e.g. such as caring for young children at night, snoring partner, shift work, etc) | .90 (±.01) | .20 (±.02) | 0.80 |
| Sleep | felt satisfied with my sleep | .91 (±.01) | .17 (±.02) | 0.83 |
| Sleep | had trouble falling or staying asleep, or waking up too early | .78 (±.02) | .40 (±.03) | 0.60 |
| Fulfilment | felt a sense of accomplishment | .80 (±.02) | .37 (±.02) | 0.63 |
| Fulfilment | felt that I am growing positively as a person | .76 (±.02) | .42 (±.03) | 0.58 |
| Fulfilment | felt like I am leading a fulfilling life | .83 (±.01) | .31 (±.02) | 0.69 |
| Fulfilment | been feeling good about myself as a person | .89 (±.01) | .20 (±.01) | 0.80 |
| Fulfilment | been feeling cheerful or bright in my mood | .84 (±.01) | .30 (±.02) | 0.70 |
| Connection | felt connected to people around me | .84 (±.01) | .30 (±.02) | 0.70 |
| Connection | felt like I have warm and trusting relationships with others | .84 (±.02) | .30 (±.03) | 0.70 |
| Connection | felt appreciated by others | .84 (±.02) | .30 (±.03) | 0.70 |
| Happiness | had little interest in people or activities that I used to enjoy | .73 (±.02) | .46 (±.03) | 0.54 |
| Happiness | been feeling down or sad in my mood | .86 (±.01) | .25 (±.02) | 0.75 |
| Happiness | found it hard to motivate myself to engage with everyday tasks | .72 (±.02) | .48 (±.03) | 0.52 |
| Happiness | felt disappointed in myself | .79 (±.02) | .37 (±.02) | 0.63 |
| Happiness | tended to get stuck in a cycle of negativity in my head | .85 (±.01) | .27 (±.02) | 0.73 |

**Table S7.** Estimated factor correlations (± standard errors) for correlated factors CFA model.

|  | Calmness | Coping | Health | Sleep | Fulfilment | Connection |
| --- | --- | --- | --- | --- | --- | --- |
| Coping | .78 (±.02) |  |  |  |  |  |
| Health | .63 (±.03) | .72 (±.02) |  |  |  |  |
| Sleep | .61 (±.03) | .55 (±.03) | .58 (±.03) |  |  |  |
| Fulfilment | .70 (±.02) | .87 (±.02) | .77 (±.02) | .58 (±.03) |  |  |
| Connection | .52 (±.03) | .68 (±.03) | .57 (±.03) | .44 (±.03) | .81 (±.02) |  |
| Happiness | .91 (±.01) | .82 (±.02) | .70 (±.02) | .57 (±.03) | .86 (±.01) | .66 (±.03) |

**Table S8.** Standardised factor loadings οnto the specific and general factors, and residual item variances (± standard errors) for bifactor CFA model. *Note: h^2^ = Item communality.*

| Factor | Item | Specific loading | General loading | Resid. var. | h^2^ |
| --- | --- | --- | --- | --- | --- |
| Calmness | found it hard to stop (or control) worrying | .45 (±.04) | .73 (±.02) | .26 (±.03) | 0.74 |
| Calmness | had difficulty switching off | .41 (±.05) | .64 (±.03) | .42 (±.04) | 0.58 |
| Calmness | noticed that my body has been tense | .45 (±.05) | .60 (±.03) | .44 (±.04) | 0.56 |
| Calmness | worried that bad things might happen to me or others close to me | .35 (±.04) | .56 (±.03) | .56 (±.03) | 0.44 |
| Coping | felt confident that I can handle problems that come my way | .35 (±.07) | .78 (±.02) | .27 (±.05) | 0.73 |
| Coping | been able to proactively manage my stress day to day | .12 (±.04) | .72 (±.02) | .47 (±.03) | 0.53 |
| Coping | felt able to cope if something unexpected happens | .51 (±.09) | .67 (±.03) | .28 (±.10) | 0.72 |
| Health | felt like I am in a good state of health | .59 (±.03) | .70 (±.02) | .16 (±.03) | 0.84 |
| Health | been managing my health well | .47 (±.03) | .71 (±.02) | .27 (±.02) | 0.73 |
| Health | felt that my physical health is not as good as I'd like it to be (given my age/life circumstances) | .48 (±.04) | .43 (±.03) | .58 (±.04) | 0.42 |
| Sleep | slept well, all things considered (e.g. such as caring for young children at night, snoring partner, shift work, etc) | .70 (±.03) | .57 (±.03) | .19 (±.03) | 0.81 |
| Sleep | felt satisfied with my sleep | .69 (±.03) | .58 (±.03) | .18 (±.02) | 0.82 |
| Sleep | had trouble falling or staying asleep, or waking up too early | .62 (±.03) | .48 (±.03) | .39 (±.03) | 0.61 |
| Fulfilment | felt a sense of accomplishment | .37 (±.03) | .73 (±.02) | .33 (±.03) | 0.67 |
| Fulfilment | felt that I am growing positively as a person | .43 (±.04) | .68 (±.02) | .35 (±.03) | 0.65 |
| Fulfilment | felt like I am leading a fulfilling life | .37 (±.04) | .76 (±.02) | .28 (±.02) | 0.72 |
| Fulfilment | been feeling good about myself as a person | .23 (±.03) | .85 (±.01) | .22 (±.01) | 0.78 |
| Fulfilment | been feeling cheerful or bright in my mood | .14 (±.03) | .82 (±.01) | .30 (±.02) | 0.70 |
| Connection | felt connected to people around me | .52 (±.03) | .66 (±.02) | .30 (±.02) | 0.70 |
| Connection | felt like I have warm and trusting relationships with others | .59 (±.03) | .62 (±.02) | .27 (±.03) | 0.73 |
| Connection | felt appreciated by others | .52 (±.03) | .64 (±.02) | .33 (±.03) | 0.67 |
| Happiness | had little interest in people or activities that I used to enjoy | .27 (±.05) | .68 (±.02) | .46 (±.03) | 0.54 |
| Happiness | been feeling down or sad in my mood | .30 (±.03) | .81 (±.02) | .26 (±.02) | 0.74 |
| Happiness | found it hard to motivate myself to engage with everyday tasks | .29 (±.04) | .67 (±.02) | .47 (±.03) | 0.53 |
| Happiness | felt disappointed in myself | .28 (±.04) | .74 (±.02) | .37 (±.03) | 0.63 |
| Happiness | tended to get stuck in a cycle of negativity in my head | .37 (±.04) | .77 (±.02) | .26 (±.03) | 0.74 |
